# Disk covering methods improve phylogenomic analyses

**DOI:** 10.1186/1471-2164-15-S6-S7

**Published:** 2014-10-17

**Authors:** Md Shamsuzzoha Bayzid, Tyler Hunt, Tandy Warnow

**Affiliations:** 1 Department of Computer Science, University of Texas at Austin, Austin, TX, 78712, USA; 2 Department of Bioengineering, The University of Illinois, Urbana, Champaign, 61801, USA

**Keywords:** multi-species coalescent process, incomplete lineage sorting, MP-EST, disk covering methods, divide-and-conquer

## Abstract

**Motivation:**

With the rapid growth rate of newly sequenced genomes, species tree inference from multiple genes has become a basic bioinformatics task in comparative and evolutionary biology. However, accurate species tree estimation is difficult in the presence of gene tree discordance, which is often due to incomplete lineage sorting (ILS), modelled by the multi-species coalescent. Several highly accurate coalescent-based species tree estimation methods have been developed over the last decade, including MP-EST. However, the running time for MP-EST increases rapidly as the number of species grows.

**Results:**

We present divide-and-conquer techniques that improve the scalability of MP-EST so that it can run efficiently on large datasets. Surprisingly, this technique also improves the accuracy of species trees estimated by MP-EST, as our study shows on a collection of simulated and biological datasets.

## Background

A standard approach to species tree estimation uses multiple loci and then concatenates alignments for each locus into a super-matrix, which is then used to estimate the species tree. When genes all evolve down the same tree topology under the same well-behaved process, then statistical methods of phylogeny estimation (such as maximum likelihood) applied to the concatenated alignment are statistically consistent, and so will return the true tree with high probability given a large enough number of sites or genes. However, when the genes evolve down different tree topologies, which can happen in the presence of gene duplication and loss, horizontal gene transfer, or incomplete lineage sorting, then there are no statistical guarantees for concatenated analyses. Furthermore, simulations have shown that concatenation can return incorrect trees with high confidence in the presence of incomplete lineage sorting [1], a population-level process modelled by the multi-species coalescent [2]. Because incomplete lineage sorting is expected to occur under many biologically realistic conditions (and especially in the presence of rapid radiations), coalescent-based species tree methods with statistical guarantees of returning the true tree with high probability (as the number of genes increases) have been developed, and are increasingly popular [3-9].

Only some of these coalescent-based methods are fast enough to be used with phylogenomic datasets that contain hundreds or thousands of genes and more than 30 or so species. For example, the fully-parametric coalescent-based methods, such as BEST [6] and *BEAST [3] that co-estimate gene trees and species trees, are limited to approximately 20 species and 100 genes (and even datasets of this size can be extremely difficult) [10,11]. The other type of coalescent-based method are called "summary methods" because they estimate species trees by combining estimated gene trees. These methods tend to be much faster than the fully-parametric methods, and some of these methods (e.g., MP-EST [5]) are able to be used with hundreds to thousands of genes.

However, even the fast summary methods can be computationally intensive on large datasets. For example, MP-EST, which has been used in many biological dataset analyses [12-15], uses a heuristic search to solve an NP-hard pseudo-maximum likelihood optimization problem (based on the triplet gene tree distribution). Our evaluation of MP-EST (reported in this paper) shows that the number of species greatly impacts the running time; thus, improving MP-EST's scalability (in terms of the number of species) is an important objective.

This paper introduces two general techniques for improving the scalability of coalescent-based species tree estimation methods so that they can analyze datasets with large numbers of species. Each technique uses an initial tree estimated on the set of species to divide the species dataset into small overlapping subsets, applies the species tree estimation method to each subset of species to produce an estimated species tree for that subset, and then combines the estimated species trees (each on a subsets of the species) into a tree on the full set of species. Furthermore, each technique can iterate, and thus return a set of candidate species trees from which the final tree is selected. The only difference between the two techniques is how the dataset is divided into subsets, with one technique using the dataset decomposition technique from DACTAL [16] and the other using a modification of the dataset decomposition technique from Rec-I-DCM3 [17].

We evaluate these two techniques on a collection of simulated and biological datasets, and show that both reduce the running time of MP-EST, one of the most popular coalescent-based summary methods. Surprisingly, these two techniques also improve the accuracy of MP-EST. Thus, the two techniques improve the scalability of MP-EST, a popular coalescent-based species tree estimation, so that it can be run on datasets with large numbers of species and provide improved topological accuracy.

## Methods

Disk-Covering Methods (DCMs) are meta-methods (employing divide-and-conquer and in some cases also iteration) designed to "boost" the performance of the existing phylogenetic reconstruction methods [17-20]. The major steps of DCMs are: (i) decomposing the dataset into overlapping subsets of taxa, (ii) estimating trees on these subsets using a preferred phylogenetic method, and finally (iii) merging the subtrees to get a tree on the full set of taxa. However, DCMs have not yet been used in the context of species tree estimation from multiple gene trees, which is the focus of this study. Although the approach we present can be used with any coalescent-based method (including ones that co-estimate gene trees and species trees, such as BEST and *BEAST), we study the technique specifically for use with MP-EST.

• Step 1: Compute a starting tree from the input set of gene trees; this is the initial guide tree (we show results using MP-EST and Matrix Representation with Parsimony (MRP) [21]).

• Step 2: Repeat for a user-specified number of iterations (we show 2 and 5).

- Step 2a: Decompose the set of species into small overlapping subsets of taxa, with target subset size specified by the user (we show 15), using the current guide tree.

- Step 2b: For each subset, create a set of gene trees by restricting the input gene trees to the species present in the subset (each such gene tree is called a subset gene tree), and then apply MP-EST to the subset gene trees to produce a newly estimated subset species tree.

- Step 2c: Combine the subset species trees estimated in Step 2b using a supertree method (we use SuperFine+MRL [22]), thus producing a tree on the full set of species. This is the new guide tree, and is used in the next iteration. We also add this tree to the set of guide trees produced during the algorithm.

• Step 3: Score each of the different guide trees produced during the algorithm with respect to the selected optimization criterion and return the tree with the best score.

We provide details for Step 2a and Step 3.

### Step 2a: dataset decomposition techniques

We explored three different techniques for decomposing the set of species into subsets: DCM1 [23], DACTAL [16], and a decomposition we call the short subtree graph (SSG) [17]. The DCM1 decomposition improved MP-EST but was less computationally efficient than the SSG-decomposition or the DACTAL-decomposition. Therefore, we focus the remainder of our discussion on the other two techniques.

*Definitions *Let *T *be an edge-weighted guide tree on the set *S *of taxa. Let *e *be an internal edge in *T*, and *t*_1_, *t*_2_, *t*_3_, *t*_4 _be the four subtrees around the edge *e *(i.e., removing *e *and its two endpoints from *T *breaks *T *into four subtrees: *t*_1_, *t*_2_, *t*_3_, *t*_4_). A *short quartet *around *e *contains four leaves, one from each of these four subtrees, where each leaf is selected to be the closest (according to the edge weights) in its subtree to *e*. Hence, the set of short quartets of a tree are obtained by taking all short quartets around all edges in the tree. We used a "padding" technique where we find a collection of closest leaves (e.g., 2 or 3, rather than just 1) from each of the four subtrees around *e*, and we call this a *padded short quartet*.

#### DACTAL-based decomposition

DACTAL uses a padded-Recursive-DCM3 decomposition (PRD), as follows. The input is a guide tree *T *(without edge weights) and target subset size *ms*. The PRD decomposition finds a "centroid" edge (i.e., an edge that splits the guide tree into two subtrees containing roughly equal numbers of leaves). The removal of this edge and its endpoints divides the tree into four subtrees, *A, B, C *and *D*. For each of these four trees, the set of at most *p*/4 (where *p *is the padding size, and *p < ms*) closest leaves to the edge *e *are selected, and put into a set *X*; four leaves selected from different subtrees around the centroid edge, using this technique, are called "padded short quartets", generalizing the concept of short quartets where only the nearest leaf in each subtree is selected [18,20]. However, if there are ties (i.e., leaves that are equally close to the branch *e*), then all leaves at the same (very close) distance are included in the set; thus, *|X| > p *is possible. Then, the set of leaves present in *A *∪ *X, B *∪ *X, C *∪ *X *and *D *∪ *X *define four overlapping subsets. If any of these sets is larger than *ms*, then the decomposition is repeated recursively on that set until all subsets have size at most *ms *and the padding size requirement is satisfied. However, if the application of the decomposition cannot reduce the subset size, then the subset is returned. Thus, both *p *and *ms *are treated as targets rather than hard constraints. For the simulated datasets studied in this paper, we set *p *= 4, which means that we only used short quartets (one leaf in each subtree around a centroid edge). However, for larger datasets, increasing *p *might lead to improved analyses.

#### SSG-based decomposition

The SSG-based decomposition technique we present in this paper is similar to the DCM3 decomposition presented in [17], but modified through the use of the "padding" (described above) so that there is more overlap between subsets.

Given input guide tree *T *and target maximum subset size (*ms*), the SSG-based decomposition creates a "padded" short subtree graph *G *= (*V, E*) as follows. First, we compute $p=\left\lfloor \frac{ms}{4}\right\rfloor$. We then compute the set of at most *p *closest leaves in each subtree around a given edge in the graph, and make a clique out of this set of (at most) 4*p *species. The graph containing all these cliques is the padded short subtree graph. Equivalently, the vertex set *V *contains the leaves in *T *(i.e., the species) and (*s_i_, s_j_*) ∈ *E *if and only if there is some edge *e *in the guide tree *T *such that *s_i _*and *s_j _*are each among the *p *nearest leaves to *e *in their respective subtrees. Because a padded short subtree graph is chordal, it contains at most *n *= *|V| *maximal cliques, and these can be found in polynomial time [24]. Note that typically the number of vertices in the maximal cliques will be at most *ms*, but some of them can be slightly bigger than *ms*. Thus, as with DACTAL, *ms *is a target maximal value, and not a strict upper bound on the size of any subset we analyze.

### Step 3: Selecting the best tree across different iterations

We explored two different optimality criteria - the maximum pseudo-likelihood score computed by MP-EST, which is based on the rooted triplet tree distribution, and a "quartet support score" [25]. The quartet support measures the similarity between a candidate tree *T *and the input gene trees, and is computed as follows. We decompose each input gene tree into its induced set of quartet trees (i.e., unrooted trees formed by picking four leaves). The quartet support score of a given candidate species tree *T *is the total, over all the input gene trees, of the number of induced quartet trees that *T *agrees with. As shown in [5], the tree that optimizes the maximum pseudo-likelihood score is a statistically consistent estimator of the true species tree under the multi-species coalescent model. Interestingly, the same is true of the quartet support score, as shown in [25].

## Experiments

We explore the performance of MP-EST [5] and these boosted versions of MP-EST on a collection of simulated and biological datasets. We compare the estimated species trees to the model species tree (for the simulated datasets) or to the scientific literature (for the biological datasets), to evaluate accuracy. The tree error is measured using the missing branch rate (also called the false negative rate), which is the percentage of the internal edges in the model tree that are missing in the estimated tree. We measure the statistical significance of the results by Wilcoxon signed-rank test with *α *= 0.05.

### Mammalian simulated datasets

We used datasets generated in another study [25] to explore performance of coalescent-based methods for estimating species trees. These datasets have gene sequence alignments generated under a multi-stage simulation process, which begins with a species tree estimated on a mammalian dataset (studied in [12]) using MP-EST, simulates gene trees down the species tree under the multi-species coalescent model (so that the gene trees can differ topologically from the species tree), and then simulates gene sequence alignments down the gene trees under the GTRGAMMA model. We direct the reader to [25] for full details.

The basic model species tree has branch lengths in coalescent units, and we produced other model species trees by rescaling the branch lengths. This rescaling varies the amount of ILS (shorter branches have more ILS), and also impacts the amount of gene tree estimation error and the average bootstrap support (BS) in the estimated gene trees. The model condition with reduced ILS was created by uniformly doubling (2X) the branch lengths, and two model conditions with higher ILS were generated by uniformly dividing the branch lengths by two (0.5X) and five (0.2X). The amount of ILS obtained without adjusting the branch lengths is referred to as "moderate ILS", and was estimated by MP-EST on the biological data. Each model species tree was then used to generate gene trees under the multi-species coalescent model. The branch lengths in the gene trees were then modified to deviate from the strict molecular clock, and sequences were simulated down each gene tree under the GTRGAMMA model.

Maximum likelihood (ML) gene trees were estimated on each sequence alignment using RAxML [26] under the GTRGAMMA model, with 200 bootstrap replicates to produce bootstrap support on the branches. The average bootstrap support (BS) in the biological data was 71%, and the sequence lengths were set to produce estimated gene trees with average BS bracketing that value - 500 bp alignments produced estimated gene trees with 63% average BS and 1000 bp alignments produced estimated gene trees with 79% average BS.

The number of genes ranged from 50 to 800 to explore both smaller and larger numbers of genes than the full biological dataset (which had roughly 400 genes). For each model condition (specified by the ILS level, the number of genes, and the sequence length), we created 20 replicate datasets.

### Biological datasets

We analyzed two biological datasets - the mammalian dataset from [12] containing 37 species and 424 genes, and the amniota dataset from [13] containing 16 species and 248 genes - using MP-EST and both versions of boosted MP-EST. We set *ms *= 15 for the mammalian dataset, and *ms *= 10 for the amniota dataset.

## Results

### Running time on simulated datasets

**Figure 1:**
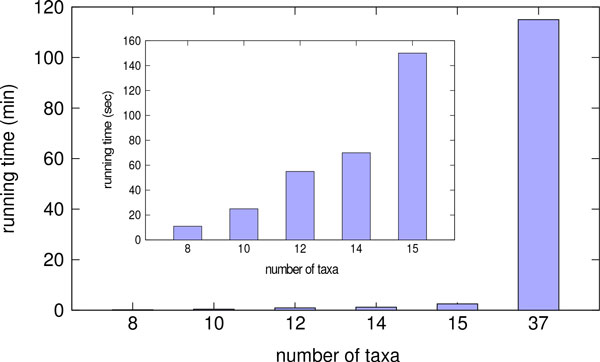
**Running time of MP-EST for varying number of taxa**. We show the running time of MP-EST on the simulated mammalian datasets for varying numbers of taxa on the model condition with moderate level of ILS, 200 genes and 500 bp sequence length. The inset subfigure shows results in seconds for 8 to 15 taxa, and the larger figure also shows results in minutes on datasets with up to 37 taxa.

Our first experiment evaluated the running time of MP-EST on different-sized subsets of the simulated mammalian datasets; see Figure 1. Note the fast increase in running time, so that MP-EST completed in 11 seconds on 8-taxon subsets, in 25 seconds on 10-taxon subsets, and in 150 seconds on 15-taxon subsets. Furthermore, MP-EST took 6900 seconds (115 minutes, or nearly two hours) to analyze the 37-taxon mammalian dataset.

In contrast, each iteration of boosted MP-EST requires much less time: 12 minutes per iteration for SSG-boosting and 7 minutes per iteration for DACTAL-boosting, each run sequentially.

**Figure 2:**
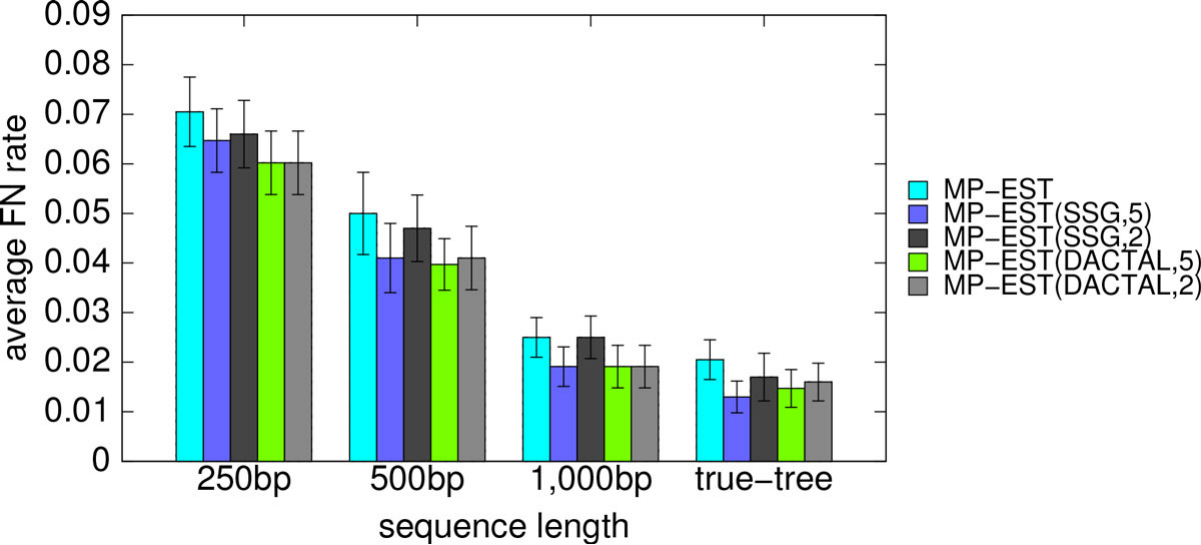
**Average FN rates of boosted MP-EST after two and five iterations**. We show the average FN rates of the best trees, with respect to the quartet support, after two and five iterations of SSG and DACTAL-based boosting on the simulated mammalian datasets with varying sequence length (200 genes, moderate amount of ILS).

The vast majority of the running time for both the DCM-boosted and SSG-boosted versions of MP-EST is in computing the starting tree (if it uses MP-EST or some other slow method) and when it runs MP-EST on subsets; all the other steps completed in seconds, run sequentially. The decomposition requires each subset to be no more than 15 species, but the average size of each subset under the SSG- and DACTAL-based decompositions was between 12 and 13; hence, MP-EST on each subset took about one minute to analyze. The number of subsets generated by the SSG-based decomposition ranged from 9 to 11, and used approximately 9-11 minutes. DACTAL decomposition typically generated only 4-5 subsets (two cases with 7 subsets), and used approximately 4-5 minutes. Thus, the DACTAL-based analysis and SSG-based analysis produced subsets of approximately the same size, but DACTAL-based analyses had generally half the number of subsets to analyze, and so took about half the time. We also observed (Figure 2 and Figs. S1 and S2 in Additional file 1) that two iterations of DACTAL-boosting achieved about the same accuracy (and sometimes better accuracy) as five iterations of SSG-boosting. Thus, DACTAL-boosting provides running time benefits compared to SSG-boosting. Finally, since using MP-EST as the starting tree is computationally expensive, we also evaluated boosting using MRP, which is a very fast method for computing the starting tree, but which is not as accurate as MP-EST for species tree estimation in the presence of ILS; see below for these results.

### Impact of boosting on topological accuracy for simulated datasets

We compared the accuracy and running time for various boosting techniques. We used MP-EST to produce the starting tree, and then ran five different iterations of DACTAL-boosting and SSG-boosting, using different subset sizes (from 15 to 22), and using different criteria (maximum pseudo-likelihood as computed by MP-EST or quartet support) to select the final tree.

**Figure 3:**
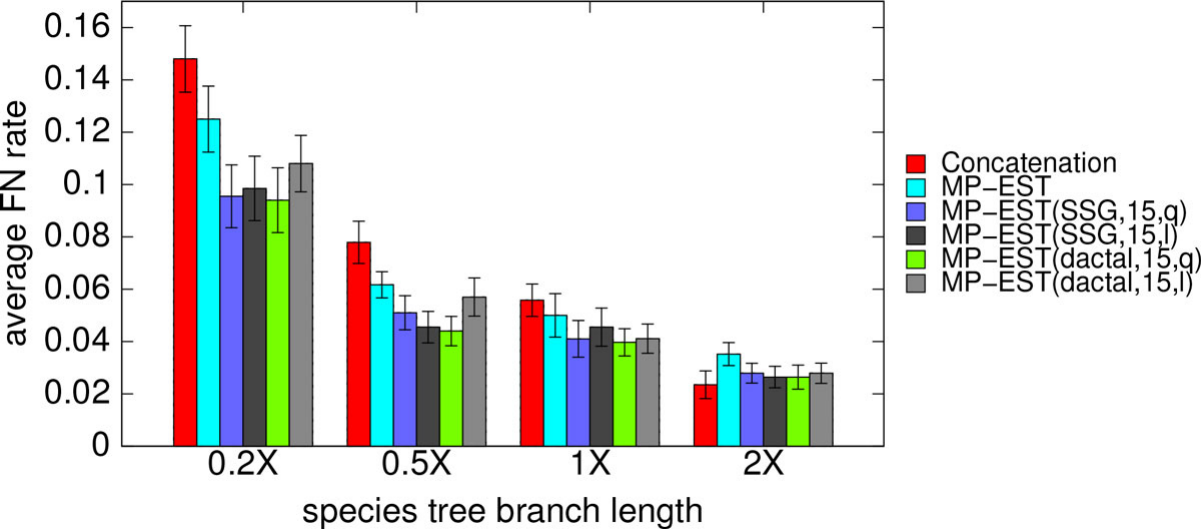
**Impact of how the final tree is selected (using quartet support or pseudo-likelihood) in boosted versions of MP-EST**. We show average FN rates of MP-EST (with and without boosting) on the simulated mammalian datasets with varying amount of ILS, using two different ways of selecting the final tree: quartet support (q) or pseudo-likelihood (l). We fixed the number of genes to 200 and sequence length to 500 bp, and varied the amount of ILS. 2X model condition contains the lowest amount of ILS while 0.2X refers to the model conditions with the highest amount of ILS. We show the results for SSG and DACTAL-based decomposition with maximum subset size 15.

As noted above, DACTAL-boosting or SSG-boosting produced the same results after five iterations. Analyses based on decompositions into subsets of size 15 completed more quickly than decompositions into larger subsets, and all subset sizes we explored (15-22) produced comparable accuracy. Finally, using quartet support scores rather than maximum pseudo-likelihood scores to select the output species tree had better overall results (Figure 3 and Figs. S5 and S6 in Additional file 1). Based on these preliminary results, we set default algorithmic parameters as follows: DACTAL decomposition, subsets of size 15, and selecting the final tree using the quartet support score. However, we show results for different combinations of the algorithmic parameters below.

**Figure 4:**
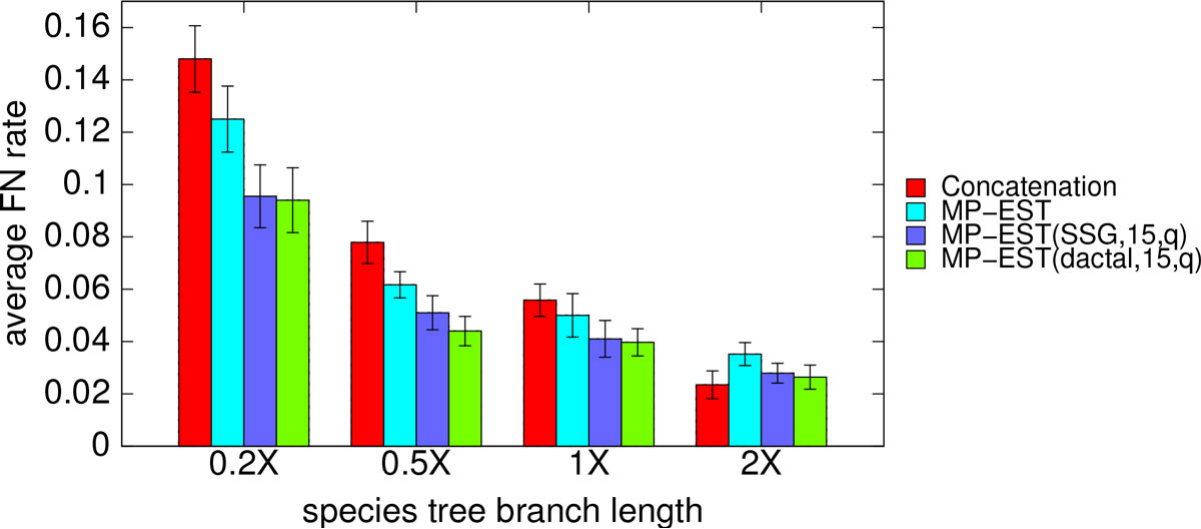
**Average FN rates of MP-EST (with and without boosting) for different levels of ILS**. Average FN rates of MP-EST (with and without boosting) over 20 replicates on the simulated mammalian datasets with varying amounts of ILS. We also show the FN rate of concatenation. We fixed the number of genes to 200 and sequence length to 500 bp, and varied the amount of ILS. 2X model condition contains the lowest amount of ILS while 0.2X refers to the model conditions with the highest amount of ILS. We show the results for short subtree graph (SSG) and DACTAL-based decompositions with maximum subset size 15. We show the FN rate of the best tree with respect to quartet support (as denoted by q in the figure legend) across five iterations.

Figure 4 shows the average FN rates of concatenation using maximum likelihood, MP-EST, and boosted MP-EST (using both DACTAL and SSG-based boosting). The results for boosting are based on starting with the MP-EST tree, then performing 5 iterations and selecting the species tree based on the quartet support. Both ways of boosting improved the accuracy of MP-EST across all levels of ILS, and were substantial on the model conditions with increased ILS (0.5X and 0.2X). We measured the statistical significance of the results using Wilcoxon signed-rank test (*p*-values given in Table S3 in Additional file 1). With the exception of the 1X model condition, the improvements of DACTAL-boosted MP-EST over un-boosted MP-EST were statistically significant (*p *values are 0.002, 0.009, 0.09 and 0.04 for 0.2X, 0.5X, 1X and 2X model conditions respectively). The improvements of SSG-boosted MP-EST over un-boosted MP-EST were statistically significant for the highest ILS level (0.2X, *p *= 0.006), but not for the other levels (*p *values were 0.13, 0.08 and 0.117 for 0.5X, 1X and 2X model conditions, respectively).

Concatenation is expected to be less accurate than coalescent-based methods when there is substantial ILS, and this is what we observed in these experiments. Thus, with the exception of the 2X model condition (which had the least ILS), concatenation was less accurate than both MP-EST and boosted MP-EST. Interestingly, the improvement of concatenation over boosted MP-EST on the 2X model condition was not statistically significant (*p *= 0.33 and *p *= 0.4 for SSG- and DACTAL-based boosting, respectively). Also, on the moderate level of ILS (1X), concatenation and MP-EST had very close performance, but boosted MP-EST was more accurate than concatenation. However, the differences between boosted MP-EST and concatenation were not statistically significant (*p *= 0.08 and *p *= 0.11 for DACTAL and SSG-based boosting respectively).

**Figure 5:**
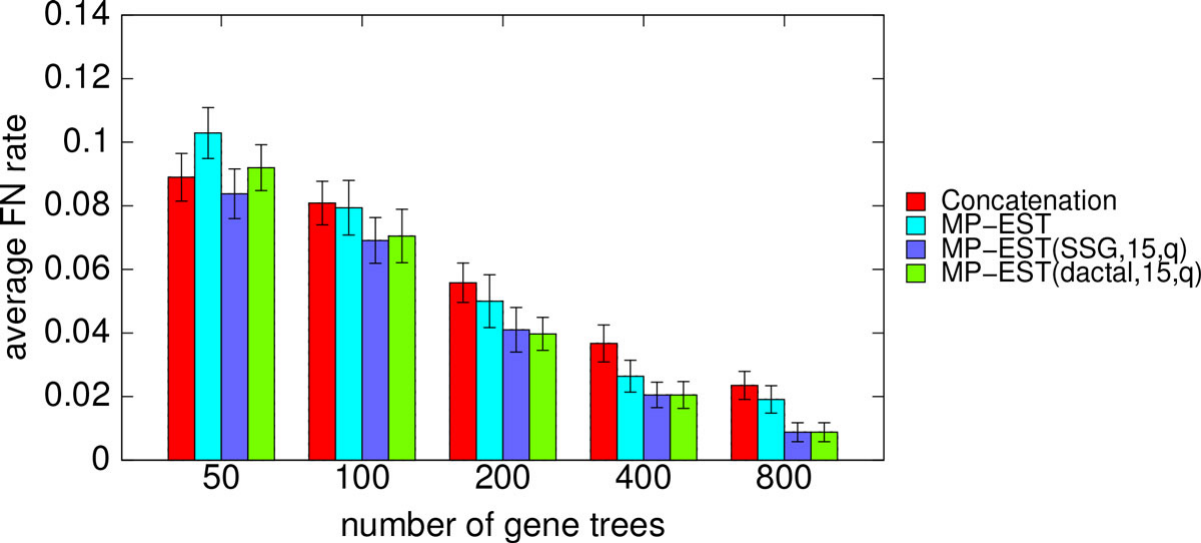
**Average FN rates of MP-EST (with and without boosting) for different number of gene trees**. Average FN rates of MP-EST (with and without boosting) over 20 replicates on the simulated mammalian datasets with varying numbers of gene trees. We also show the FN rate of concatenation. We varied the number of genes from 100 to 800, and set the amount of ILS to 1X level and the sequence length to 500 bp. We show the results for short subtree graph (SSG) and DACTAL-based decompositions with maximum subset size 15. We show the FN rate of the best tree with respect to quartet support (as denoted by q in the figure legend) across five iterations.

Figure 5 shows the comparison between unboosted and boosted MP-EST using both SSG- and DACTAL-based decomposition on the simulated mammalian datasets with 50 to 800 genes, moderate levels of ILS (1X), and sequence length set to 500 bp. Both SSG and DACTAL-based decomposition improved MP-EST in all cases, sometimes substantially. The improvements of SSG-based boosting over un-boosted MP-EST were statistically significant except for the 200- and 400-gene cases (*p *values were 0.003, 0.02, 0.08, 0.09, and 0.01 for model conditions with 50, 100, 200, 400, and 800 genes, respectively). DACTAL-based boosting was significantly better than un-boosted MP-EST on the 800-genes case but not on the others (*p *values were 0.06, 0.09, 0.09, 0.09 and 0.01 for model conditions with 50, 100, 200, 400, and 800 genes, respectively).

The comparison between concatenation and (boosted) MP-EST is also interesting. For the 50-gene case, concatenation was more accurate than unboosted MP-EST, but DACTAL-boosted MP-EST matched the accuracy of concatenation, and SSG-boosted MP-EST was slightly more accurate than concatenation. For other cases (100-800 genes), the differences between concatenation and MP-EST were not statistically significant (*p >*0.05), but both SSG-boosted and DACTAL-boosted versions of MP-EST were more accurate than concatenation. Furthermore, the improvement of boosted MP-EST over concatenation was statistically significant for 400- and 800-gene cases (*p *= 0.02 and 0.008 for the 400- and 800-gene cases, respectively, for both SSG and DACTAL-based boosting).

**Figure 6:**
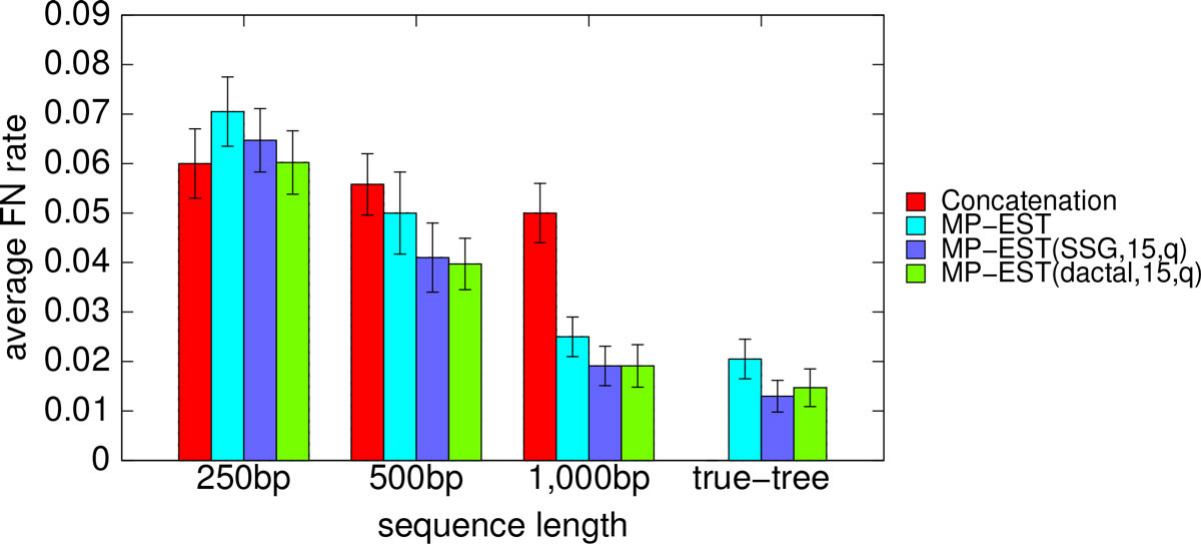
**Average FN rates of MP-EST (with and without boosting) for different sequence lengths**. Average FN rates of MP-EST (with and without boosting) over 20 replicates on the simulated mammalian datasets with different amounts of gene tree estimation error by varying the sequence lengths. We also show the FN rate of concatenation. We varied the sequence lengths from 250 bp to 1000 bp with 200 genes and moderate amount of ILS (1X). We show the results for short subtree graph (SSG) and DACTAL-based decompositions with maximum subset size 15. We show the FN rate of the best tree with respect to quartet support (as denoted by q in the figure legend) across five iterations.

Figure 6 compares boosted and un-boosted MP-EST on the mammalian datasets with varying sequence lengths. We fixed the amount of ILS to the moderate level (1X) and number of genes to 200, and varied the sequence lengths from 250 bp to 1000 bp. We also show the results on true gene trees (i.e., without estimation error). Boosting improved the accuracy of MP-EST in all cases. The improvements were statistically significant for the 250 bp case with DACTAL-based boosting and on the true trees for both types of boosting (*p <*0.05). On the 250 bp condition (which has the highest gene tree estimation error) concatenation was more accurate than MP-EST, and boosted MP-EST matched concatenation.

### Results on biological datasets

*Amniota dataset*. We analyzed data for 248 genes on 16 amniota species from Chiari et al. [13]. Previous studies had placed turtles as the sister to birds and crocodiles (Archosaurs) [27-29]. Chiari et al. [13] used concatenation and MP-EST with multi-locus bootstrapping on two sets of gene trees - one based on amino acid (AA) and the other based on nucleotide (NT) alignments. Concatenation and MP-EST on the AA gene trees resolved the clade as (turtles,(birds, crocodiles)) (i.e., birds and crocodiles were considered sister taxa, consistent with the earlier studies) while MP-EST on the NT data produced (birds,(turtles,crocodiles)), and so contradicted the previous studies. Because the concatenation tree and the MP-EST(AA) tree agreed and were consistent with previous studies, the resolution with turtles as sister to birds and crocodiles was considered more likely to be correct.

**Figure 7:**
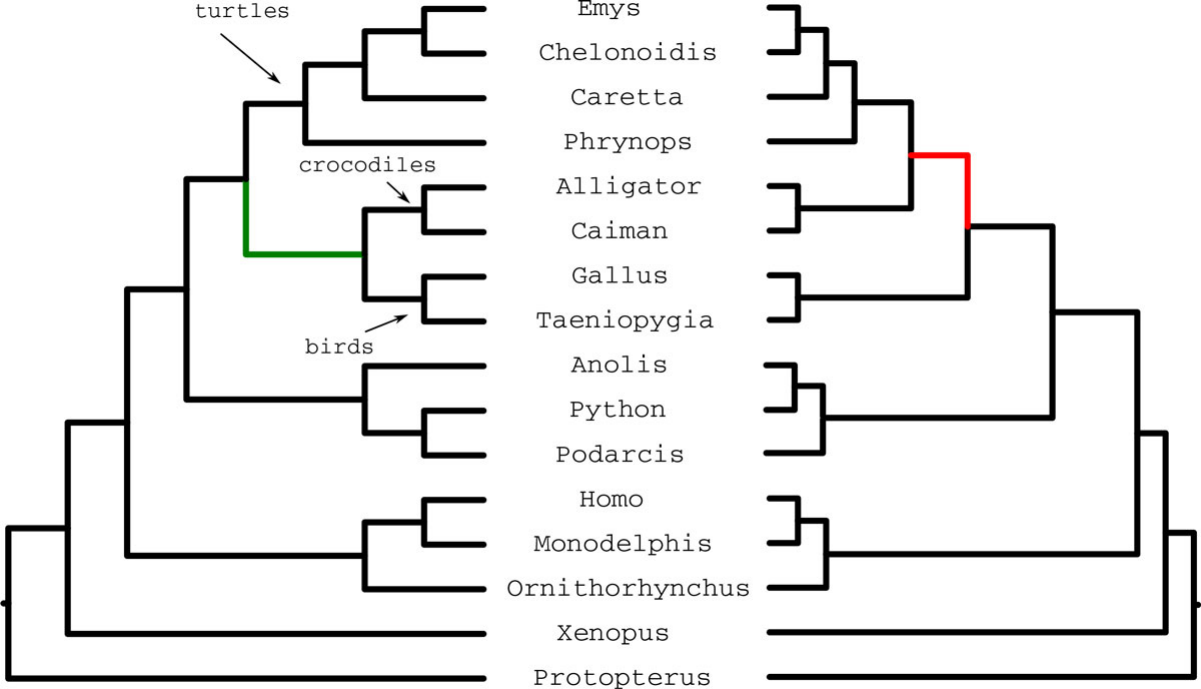
**Analyses of the amniota dataset using MP-EST (with and without boosting)**. We show the trees estimated by MP-EST (right) and SSG and DACTAL-boosted MP-EST (left) using the MP-EST and MRP-estimated starting tree on the nucleotide amniota dataset from [13]. The sister relationship of crocodiles and birds is considered reliable, and is recovered in the SSG-boosted MP-EST tree. However, the MP-EST analysis of this dataset places crocodiles as sister to turtles (indicated by the red edge), and is not considered reliable.

We ran MP-EST on the NT datasets containing 248 gene trees with 10 independent runs and retained the tree with the highest maximum likelihood value; this produced the same tree reported in [13]. We then ran four versions of boosted MP-EST, with SSG- and DACTAL-based decompositions, and using the MP-EST starting tree. For each analysis, we ran five iterations and retained the tree with the highest quartet support across the five iterations. All variants produced the same tree, resolving Archosaurs as (turtles,(birds,crocodiles)) (Figure 7). Thus, the boosted MP-EST trees were consistent with concatenation and other previous studies.

*Mammalian dataset*. Song *et al*. [12] analyzed a dataset with 447 genes across 37 mammalian species using MP-EST and concatenation. In our analysis of this data we detected 21 genes with mislabelled sequences (incorrect taxon names, confirmed by the authors) which we removed from the dataset. We also identified two additional gene trees that were clearly topologically very different from all other gene trees, and removed these as well. We ran MP-EST on the 424 gene trees with SSG and DACTAL-based boosting using the MP-EST starting tree. All analyses we ran produced the same tree (see Fig. S9 in Additional file 1).

### Pseudo-likelihood scores and quartet support values

Our analyses of the simulated and biological datasets showed that MP-EST always found trees with pseudo-likelihood scores that were at least as good as those found by any boosted MP-EST analysis, over all the iterations. In other words, the best pseudo-likelihood score was always found in the MP-EST starting tree. On the other hand, the best quartet support score was nearly always found in a subsequent iteration, for both types of boosting techniques. The first of these observations suggests that MP-EST is doing a reasonably good job of solving its optimization problem, since boosting is not improving its search. The second of the observations is also very interesting, since the boosting techniques are not explicitly designed to optimize quartet support, and we have no explanation for this trend.

### Robustness to the starting trees

In the experiments shown so far, the starting tree was produced using MP-EST. We tested robustness to the starting tree by using MRP, a fast supertree technique, to compute a starting tree. However, unlike MP-EST, MRP has not been shown to be statistically consistent in the presence of ILS, and so is not likely to be as accurate as MP-EST

**Figure 8:**
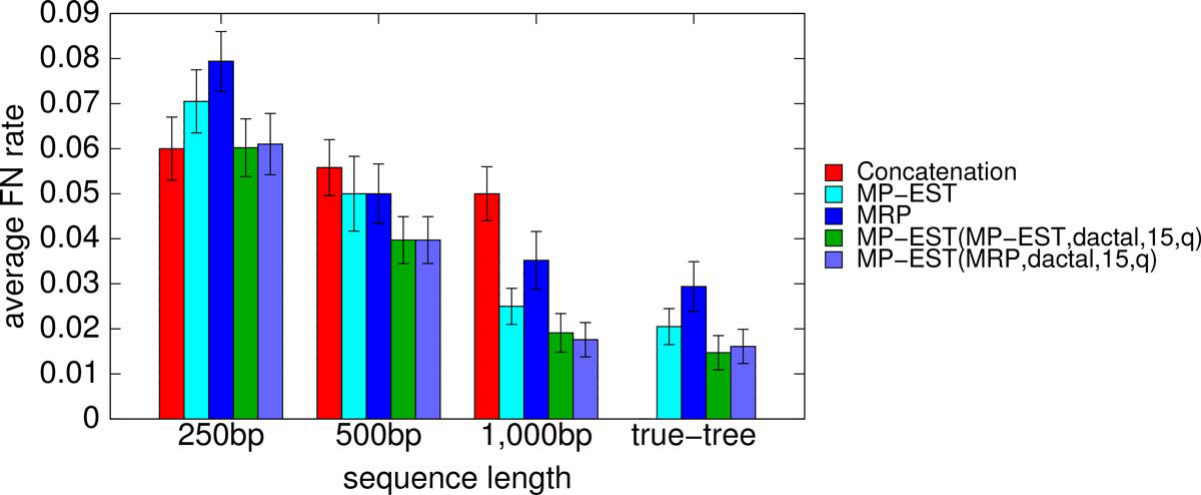
**Impact of different starting trees on DACTAL-based boosting with MP-EST**. We show the average FN rates of the best trees, with respect to the quartet support, after five iterations of DACTAL-based boosting using MP-EST and using the starting trees estimated by MRP and MP-EST on the simulated mammalian datasets with varying sequence length (200 genes, moderate amount of ILS). We ran MP-EST on the subsets produced by the DACTAL-based decomposition with maximum subset size 15 using different starting trees. MP-EST(MRP,dactal,15,q) refers to the results obtained by using the MRP-estimated starting tree, while MP-EST(MP-EST,dactal,15,q) refers to the results obtained by using the starting tree estimated by MP-EST. We also show the FN rates of concatenation and the starting trees estimated by MP-EST and MRP.

Analyses of all biological datasets produced the same results, whether based on MRP or MP-EST starting trees. Results on the simulated datasets (Figure 8 and Figs. S3, S4 in Additional file 1) show that MRP starting trees were generally not as accurate as MP-EST starting trees, but that five iterations of DACTAL-boosting from either starting tree produced essentially the same level of accuracy.

### Statistical consistency

The following theorem is a direct corollary of Theorem 1 in [16].

**Theorem 1: ***Let T be the true species tree, and let S_1_, S_2_,..., S_k _be the subsets created by a DACTAL- or SSG-decomposition with T as the starting tree. Let t_i _be the true species tree on S_i_, i = 1, 2,..., k. Then the Strict Consensus Merger (and by extension also SuperFine+MRL), applied to the set t_1_, t_2_,..., t_k, _will return the species tree T*.

Comment: SuperFine+MRL has two steps: first it computes the Strict Consensus Merger (SCM), and then it resolves high degree nodes in the SCM tree using MRL. Therefore, if SCM produces a fully resolved tree, SuperFine+MRL returns the SCM tree.

Therefore, the following corollary can be easily proven:

**Corollary 1: ***If the starting tree is computed using a method that is statistically consistent under the multi-species coalescent model, then the pipeline based on either the DACTAL or SSG decomposition is statistically consistent under the multi-species coalescent model*.

## Discussion

The results shown in this study suggest that using iteration and divide-and-conquer (within the DACTAL-based and SSG-based decomposition techniques) improved the topological accuracy of MP-EST. Furthermore, the specific choice of dataset decomposition technique (DACTAL-based or SSG-based) had little impact on accuracy. The improvement obtained by selecting trees based on their quartet support scores instead of their maximum pseudo-likelihood scores is very interesting, and suggests the possibility that although both optimality criteria are statistically consistent ways of searching for species trees under the multi-species coalescent, the quartet support score might have better empirical performance than the pseudo-likelihood score, at least under some conditions.

While most of the analyses were based on using MP-EST to produce the starting tree, we also showed that using MRP (a supertree method) to produce the starting tree resulted in comparable accuracy after five iterations. Since MRP generally produced less accurate starting trees than MP-EST, this suggests that the boosting techniques are robust to the starting tree. Furthermore, MRP was very fast on these datasets, completing in just ten seconds. Thus, when used with MRP as a starting tree, the entire pipeline (computing the starting tree, running five iterations of DACTAL boosting, and selecting the final tree) completes in 35 minutes. By comparison, MP-EST run without boosting takes nearly 115 minutes (nearly two hours). Thus, boosting improves the speed of MP-EST. If we use SuperFine+MRL or SuperFine+MRP to compute the starting tree, then DACTAL-boosted MP-EST should be fast, even for large numbers of species, since computing the starting tree using SuperFine is typically very fast, even on large datasets [22]. Furthermore, although we do not explore datasets with more than 37 species, the running times in Figure 1 suggest that MP-EST may be computationally infeasible for datasets with a few hundred species. By contrast, boosted versions of MP-EST are likely to scale close to linearly with the number of species, and are embarrassingly parallel. Thus, large-scale analyses of even several hundred species should be feasible using boosted MP-EST.

While the improvement in speed was expected, the improvement in accuracy was unexpected, and merits discussion. One possibility is that the performance we observed is mainly the result of some specific property of the simulation conditions we explored in this study, and that a larger study might show a difference in relative performance between boosted and unboosted MP-EST. However, both boosted versions of MP-EST gave more accurate results on the biological amniota dataset, and so that is not likely to be the answer. As noted, MP-EST is a heuristic for maximum pseudo-likelihood, and so another possible explanation is that MP-EST might have difficulty finding good solutions to its optimization problem on large datasets. However, the trees found by MP-EST had ML scores that were at least as good (and most often better) than the trees produced in any iteration by the boosted versions of MP-EST. Thus, this was clearly not the reason boosting improves MP-EST.

Instead, the data suggests that the boosting technique leads to trees with better quartet support scores, and that using quartet support scores to select the best species tree might be helping these boosted versions of MP-EST to produce more accurate trees. This hypothesis is supported by the fact that selecting the best tree based on the quartet support produced improved topological accuracy compared to selecting the best tree based on the pseudo-likelihood score, and that the quartet support optimization criterion is statistically consistent under the multi-species coalescent model [25].

## Conclusions

MP-EST is one of the popular methods for estimating species trees from a collection of gene trees, and has statistical guarantees under the multi-species coalescent model. MP-EST is fast on small datasets (with not too many species) but its running time grows quickly with the number of species. We presented two iterative divide-and-conquer techniques (DACTAL-boosting and SSG-boosting) to use with MP-EST, with the goal of enabling MP-EST to analyze datasets with large numbers of species more efficiently. We tested these techniques on a collection of simulated and biological datasets, and showed that boosted versions of MP-EST were fast and highly accurate using these divide-and-conquer methods. The improvement in accuracy obtained by using these boosting techniques is not explained by any failure in MP-EST to optimize maximum likelihood effectively, but rather suggests the possibility that an alternative optimization criterion - quartet support - may be a highly effective approach to estimating species trees under the multi-species coalescent model.

## Methods and commands

*Gene tree estimation: *RAxML version 7.3.5 [30] was used to estimate gene trees under the GTRGAMMA model, using the following command:

raxmlHPC-SSE3 -m GTRGAMMA -s [input alignment] -n [output name] -N 20 -p [random seed number]

The following command was used for bootstrapping:

raxmlHPC-SSE3 -m GTRGAMMA -s [input alignment] -n [output name] -N 200 -p [random seed number] -b [random seed number]

*Concatenation: *For the concatenated analysis, we computed a parsimony starting tree using RAxML version 7.3.5, and then ran RAxML-light version 1.0.6. We used the following commands:

raxmlHPC-SSE3 -y -s supermatrix.phylip -m GTRGAMMA -n [output name] -p [random seed number]

raxmlLight-PTHREADS -T 4 -s supermatrix.phylip -m GTRGAMMA -n name -t [parsimony tree]

*MP-EST: *We used version 1.3 of MP-EST.

*MRP: *We created MRP matrices using a custom Java program, and solved MRP heuristically using the default approach available in PAUP^* ^(v. 4. 0b10) [31]. PAUP^* ^generates an initial tree through random sequence addition and then performs Tree Bisection and Reconnection (TBR) moves until it reaches a local optimum. This process is repeated 1000 times, and at the end the most parsimonious tree is returned. When multiple trees are found with the same maximum parsimony score, the "extended majority consensus" of those trees is returned. See Additional file 1 for the PAUP^* ^commands used to run MRP.

*Decompositions and SuperFine: *We used our custom scripts written in various languages (Perl, Python, C++ and Java) for SSG and DACTAL-based decomposition and SuperFine.

## Competing interests

The authors declare that they have no competing interests.

## Authors' contributions

TW proposed the study, helped designed the study, and helped write the paper. MSB helped design the study, implemented the SSG and DACTAL decompositions, performed the analyses, and helped write the paper. TH implemented DCM1-based decomposition and performed analyses. All authors read and approved the final manuscript.

## Supplementary Material

Additional file 1 Click here for file

## Declarations

This work was partially supported by National Science Foundation grants DEB 0733029 and DBI 1062335 to TW.

This article has been published as part of *BMC Genomics *Volume 15 Supplement 6, 2014: Proceedings of the Twelfth Annual Research in Computational Molecular Biology (RECOMB) Satellite Workshop on Comparative Genomics. The full contents of the supplement are available online at http://www.biomedcentral.com/bmcgenomics/supplements/15/S6.
