# Supplementary material for: Disk covering methods improve phylogenomic analyses
Source: BMC Genomics. 2014 Oct 17;15(Suppl 6):S7. doi: 10.1186/1471-2164-15-S6-S7 (PMC4239662; doi:10.1186/1471-2164-15-S6-S7)
Supplement: Additional file 1 [file 1471-2164-15-S6-S7-S1.pdf]

Supplementary Material for
Disk Covering Methods Improve Phylogenomic Analyses

Md Shamsuzzoha Bayzid, Tyler Hunt and Tandy Warnow

September 10, 2014

1 Additional figures and tables

Additional figures and tables omitted from the main paper due to space constraints are presented here.

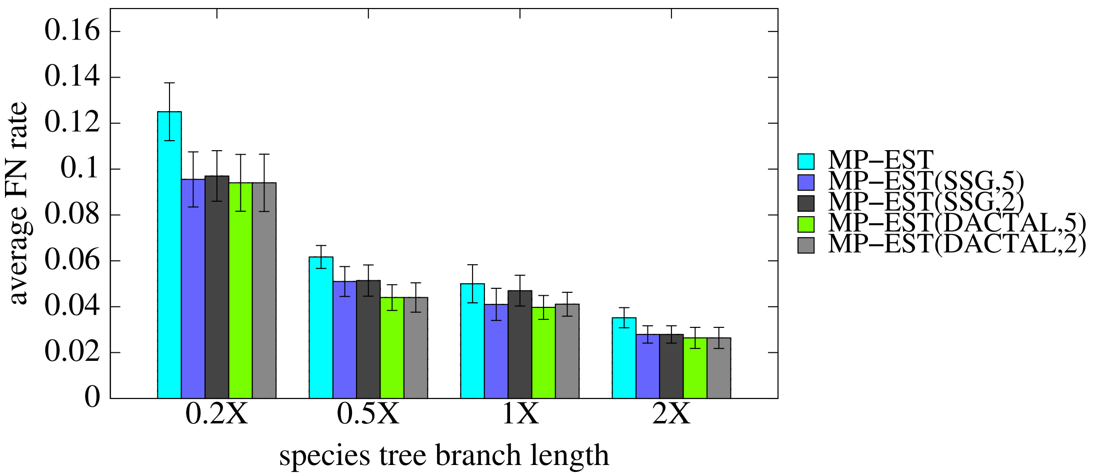

Figure S1: **Average FN rates of boosted MP-EST after two and five iterations.** We show the average FN rates of the best trees, with respect to the quartet support, after 2 and 5 iterations of SSG and DACTAL-based boosting on the simulated mammalian datasets with varying amount of ILS (200 genes, 500bp).

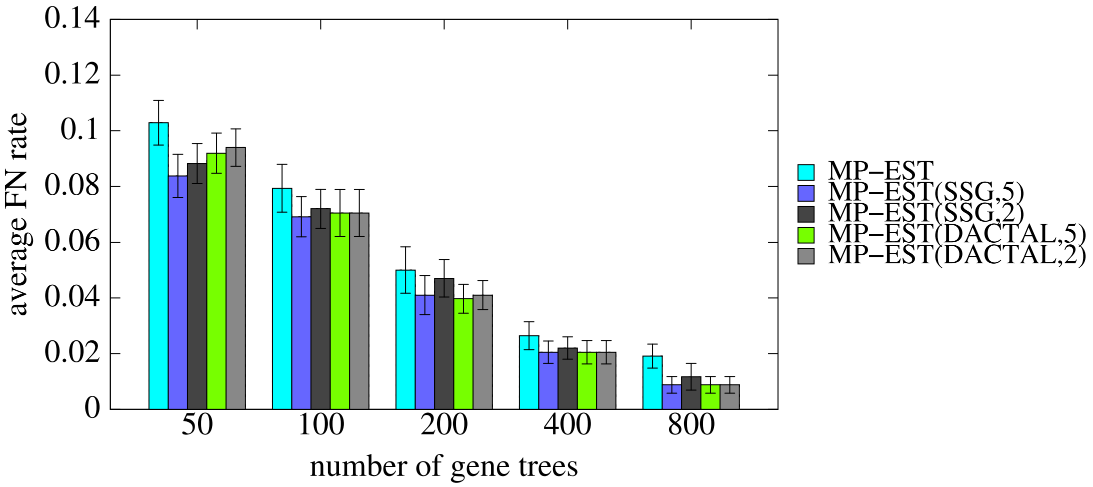

Figure S2: **Average FN rates of boosted MP-EST after two and five iterations.** We show the average FN rates of the best trees, with respect to the quartet support, after 2 and 5 iterations of SSG- and DACTAL-based boosting on the simulated mammalian datasets with varying numbers of gene trees (moderate amount of ILS, 500bp).

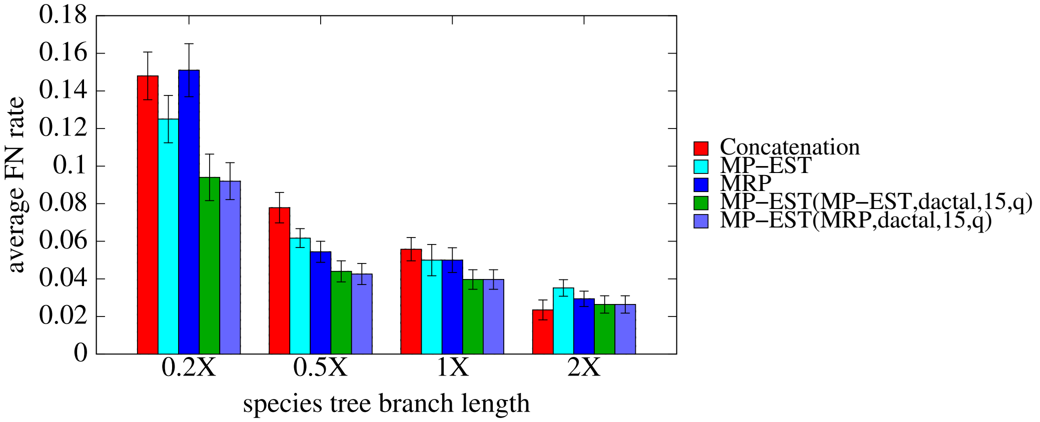

Figure S3: **Impact of different starting trees on DACTAL-based boosting with MP-EST.** We show the average FN rates of the best trees, with respect to the quartet support, after 5 iterations of DACTAL-based boosting using MP-EST and using the starting trees estimated by MRP and MP-EST on the simulated mammalian datasets with varying amount of ILS (200genes and 500bp). We ran MP-EST on the subsets produced by DACTAL-based decomposition with maximum subset size 15 using different starting trees. MP-EST(MRP,dactal,15,q) refers to the results obtained by using the MRP-estimated starting tree, while MP-EST(MP-EST,dactal,15,q) refers to the results obtained by using the starting tree estimated by MP-EST. We also show the FN rates of concatenation and the starting trees estimated by MP-EST and MRP.

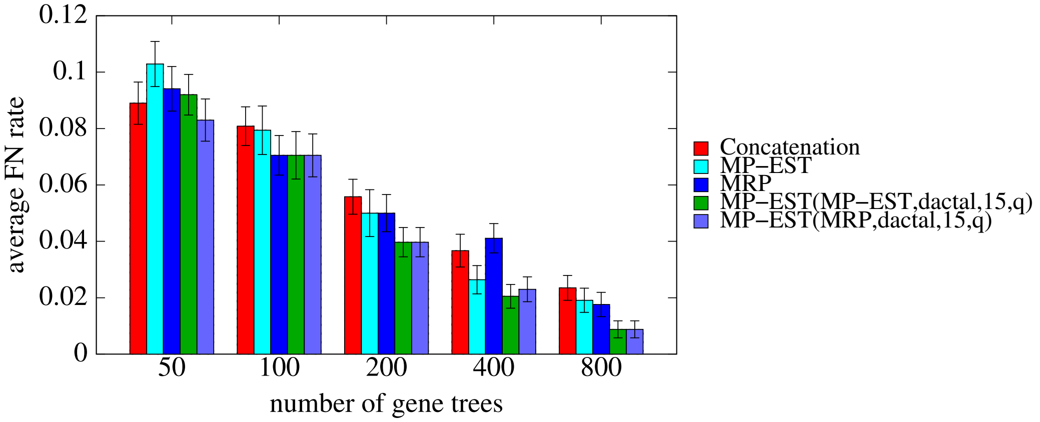

Figure S4: **Impact of different starting trees on DACTAL-based boosting with MP-EST.** We show the average FN rates of the best trees, with respect to the quartet support, after 5 iterations of DACTAL-based boosting using MP-EST and using the starting trees estimated by MRP and MP-EST on the simulated mammalian datasets with varying numbers of genes (500bp, moderate amount of ILS). We ran MP-EST on the subsets produced by DACTAL-based decomposition with maximum subset size 15 using different starting trees. MP-EST(MRP,dactal,15,q) refers to the results obtained by using the MRP-estimated starting tree, while MP-EST(MP-EST,dactal,15,q) refers to the results obtained by using the starting tree estimated by MP-EST. We also show the FN rates of concatenation and the starting trees estimated by MP-EST and MRP.

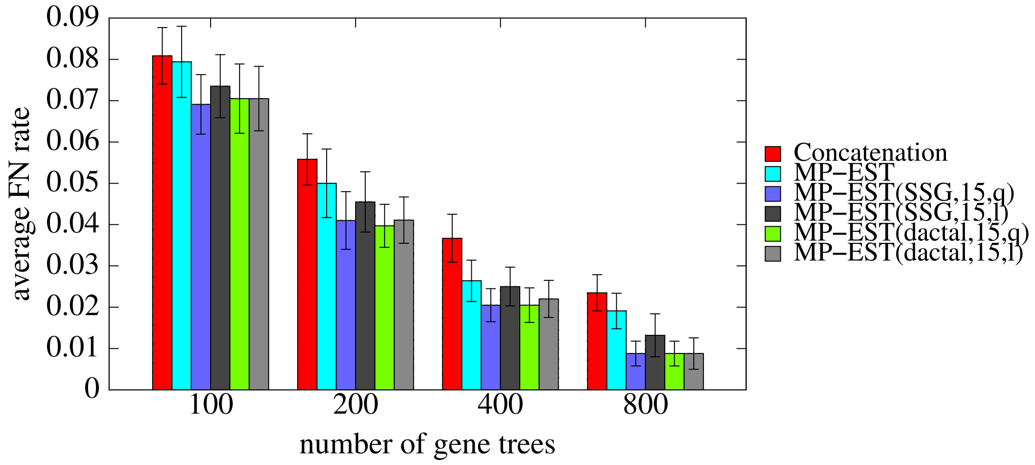

Figure S5: **Impact of how the final tree is selected (using quartet support or pseudo-likelihood) in boosted versions of MP-EST.** We show average FN rates of MP-EST (with and without boosting) on the simulated mammalian datasets with varying numbers of gene trees, using two different ways of selecting the final tree: quartet support (q) or pseudo-likelihood (l). We fixed the amount of ILS to moderate level (1X) and sequence length to 500bp, and varied the number of genes from 100 to 800. We show the results for SSG- and DACTAL-based decompositions with maximum subset size 15.

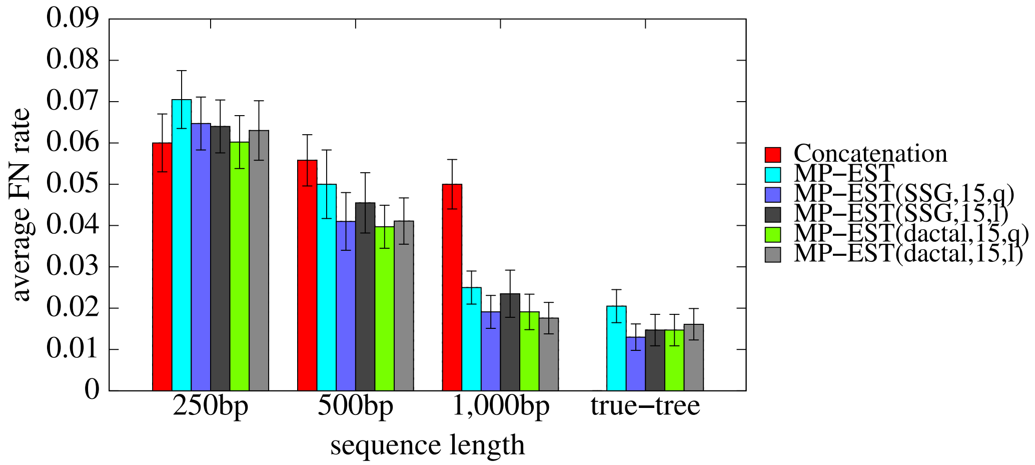

Figure S6: **Impact of how the final tree is selected (using quartet support or pseudo-likelihood) in boosted versions of MP-EST.** We show average FN rates of MP-EST (with and without boosting) on the simulated mammalian datasets with varying numbers of gene trees, using two different ways of selecting the final tree: quartet support (q) or pseudo-likelihood (l). We fixed the amount of ILS to moderate level (1X) and number of genes to 200, and varied the sequence lengths from 250bp to 1000bp. We show the results for SSG- and DACTAL-based decompositions with maximum subset size 15.

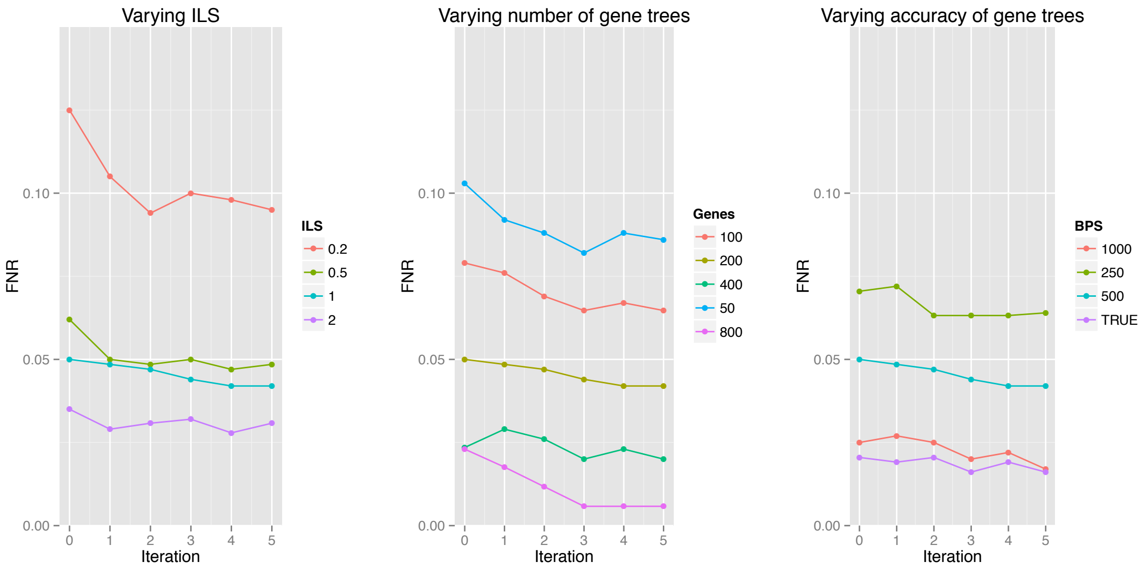

Figure S7: **Average FN rates of MP-EST with SSG-based boosting across five iterations.** We show the average FN rates of MP-EST (over 20 replicates) with SSG-based boosting accross 5 iterations on the various model conditions of the simulated mammalian datasets. Iteration 0 represents the FN rate of the initial guide tree estimated by MP-EST.

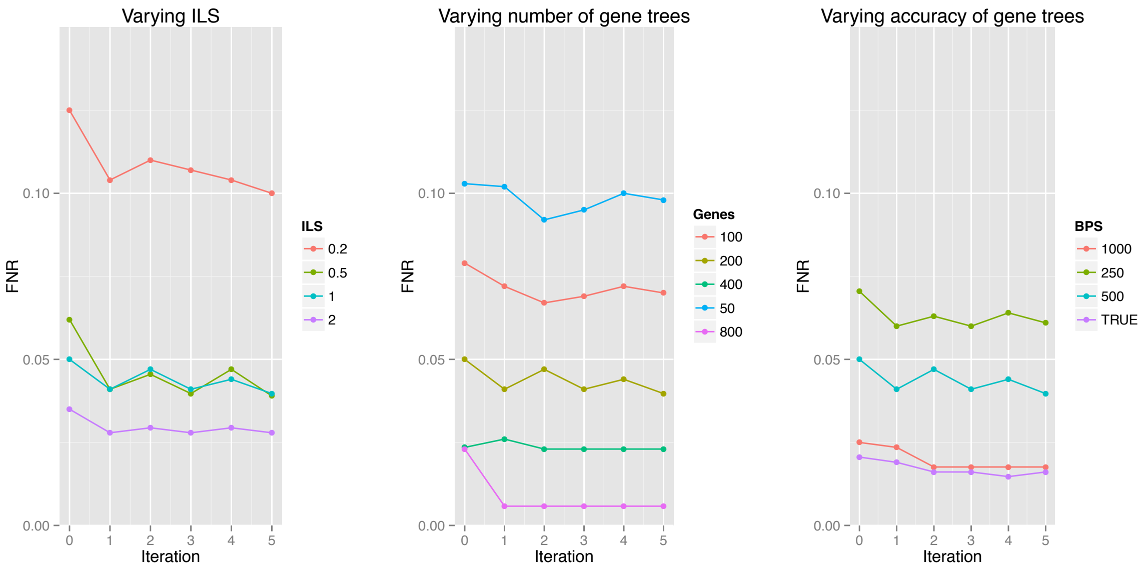

Figure S8: **Average FN rates of MP-EST with DACTAL-based boosting across five iterations.** We show the average FN rates of MP-EST (over 20 replicates) with DACTAL-based boosting accross 5 iterations on the various model conditions of the simulated mammalian datasets. Iteration 0 represents the FN rate of the initial guide tree estimated by MP-EST.

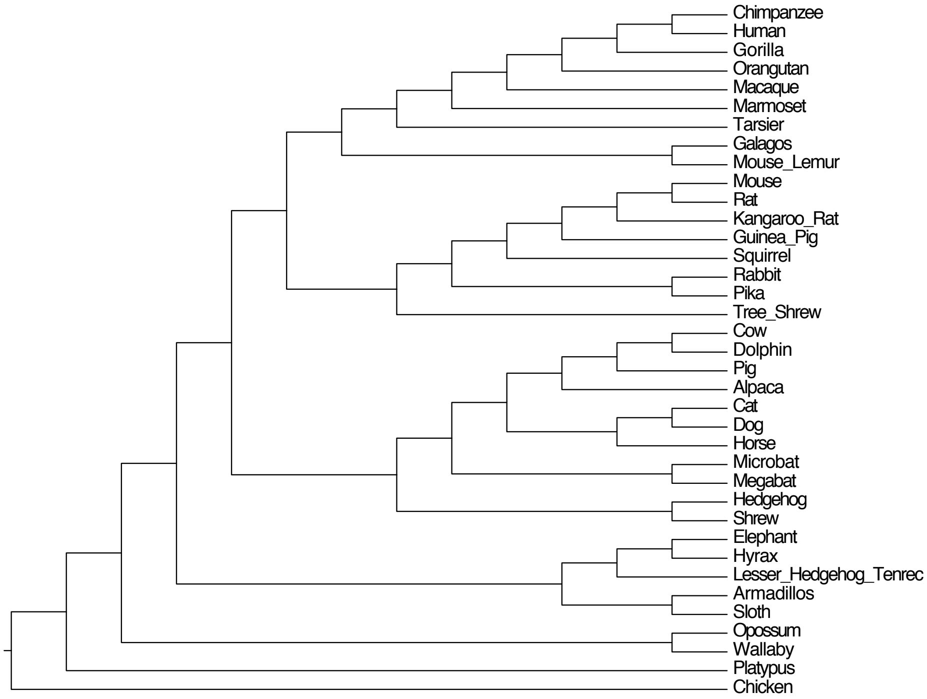

Figure S9: **Analyses of the mammalian dataset using MP-EST (with and without boosting).** MP-EST with SSG- and DACTAL-based boosting using both MP-EST and MRP-estimated starting tree produced the same tree as un-boosted MP-EST.

Table S1: Average log likelihood values (over 20 replicates) for different species trees. We estimated the log likelihood values using MP-EST. We show the likelihood values for the initial tree estimated by MP-EST and the true species tree (which is also estimated by MP-EST from the biological datasets.) For SSG and DACTAL-based boosting, we find the best tree across the five iterations with respect to the log likelihood value estimated by MP-EST with branch length optimization. The best likelihood values are shown in bold.

	Log-likelihood values			
Model condition	starting tree	best tree (SSG)	best tree (DACTAL)	model tree
0.2X,200gt,500bp	**-1338135**	-1458629	-1477619	-1338257
0.5X,200gt,500bp	**-1001218**	-1161407	-1280063	-1001269
1X,200gt,500bp	**-745312**	-903602	-1145433	-745342
2X,200gt,500bp	**-613190**	-808855	-782705	-613215
1X,100gt,500bp	**-370058**	-437613	-469161	-370154
1X,400gt,500bp	**-1486055**	-1952924	-1717131	-1486067
1X,800gt,500bp	**-2969112**	-3721911	-3407789	-2969119
1X,200gt,250bp	**-999941**	-1119309	-1145887	-1000048
1X,200gt,1000bp	**-563889**	-778484	-703988	-563904
1X,200gt,true gene tree	**-465251**	-755994	-628703	-465264

Table S2: Average quartet supports of different species trees. We show the average (over 20 replicates) number of satisfied quartets (in the input gene trees) by different species trees for various model conditions. For SSG and DACTAL-based boosting, we find the best tree across the five iterations with respect to the number of satisfied quartets. The best quartet support values are shown in bold.

	Quartet support			
Model condition	MP-EST	SSG	DACTAL	true species tree
0.2X,200gt,500bp	7818695	7819738	**7820187**	7816140
0.5X,200gt,500bp	10004913	10006656	**10006864**	10003929
1X,200gt,500bp	11269452	**11269960**	10006835	11266716
2X,200gt,500bp	11944097	11944516	**11944554**	11943759
1X,100gt,500bp	5635460	**5635757**	5635491	5630260
1X,400gt,500bp	22533544	22534293	**22534313**	22531906
1X,800gt,500bp	45095970	**45096812**	42841193	45096639
1X,200gt,250bp	10559948	**10560603**	10560467	10557321
1X,200gt,1000bp	11585974	**11586449**	11586514	11584969
1X,200gt,true gene tree	11745969	**11746174**	11746169	11744078

Table S3: p-values measured by Wilcoxon signed-rank text for the simulated mammalian datasets. We evaluate the statistical significance of differences in species tree topology using Wilcoxon signed-rank test with $\alpha = 0.05$. We show the p-values indicating whether the differences between two methods are statistically significant. We compare concatenation (CA) and MP-EST (unboosted) with SSG and DACTAL-boosted MP-EST.

	p-values				
Model condition	CA vs. MP-EST	MP-EST vs. MP-EST (SSG)	MP-EST vs. MP-EST (DACTAL)	CA vs. MP-EST (SSG)	CA vs. MP-EST (DACTAL)
0.2X,200gt,500bp	0.014	0.006	0.002	0.0002	0.0001
0.5X,200gt,500bp	0.03	0.13	0.009	0.01	0.003
1X,200gt,500bp	0.433	0.08	0.09	0.11	0.08
2X,200gt,500bp	0.06	0.117	0.04	0.33	0.45
1X,50gt,500bp	0.023	0.003	0.06	0.41	0.31
1X,100gt,500bp	0.39	0.02	0.09	0.1	0.16
1X,400gt,500bp	0.08	0.09	0.09	0.02	0.02
1X,800gt,500bp	0.27	0.01	0.01	0.008	0.008
1X,200gt,250bp	0.22	0.18	0.01	0.27	0.49
1X,200gt,1000bp	0.0004	0.1	0.06	0.0002	0.0004
1X,200gt,true gene tree	NA	0.03	0.06	NA	NA

Table S4: Average number of subsets and subset sizes for SSG and DACTAL-based decomposition. We show the average (over 20 replicates) number of subsets and average subset sizes obtained from SSG and DACTAL-based decomposition, in the first iteration, for various model conditions of the simulated mammalian datasets.

	SSG		DACTAL	
Model condition	# subset	subset size	# subset	subset size
0.2X,200gt,500bp	10.6	12.5	7.3	13
0.5X,200gt,500bp	10.3	12.6	7.35	12.8
1X,200gt,500bp	10.35	12.5	4.35	12.2
2X,200gt,500bp	11	12.5	4	13
1X,100gt,500bp	10.55	12.6	4.2	12.5
1X,400gt,500bp	10.35	12.5	4.1	12.7
1X,800gt,500bp	9.65	12.4	4	13
1X,200gt,250bp	11	12.7	4.05	12.9
1X,200gt,1000bp	9.8	12.6	4.1	12.7
1X,200gt,true gene tree	10.1	12.7	4.15	12.1

Methods and commands

We solved MRP heuristically using the default approach available in PAUP*. Below are the PAUP* commands used.

```
begin paup;
set criterion=parsimony maxtrees=1000
increase=no;
hsearch start=stepwise addseq=random
nreps=100 swap=tbr;
filter best=yes;
savetrees file = <treeFile> replace=yes format=altnex;
contree all/ strict=yes
treefile = <strictConsensusTreeFile>
replace=yes;
tcontree all/ majrule=yes strict=no
treefile = <majorityConsensusTreeFile>
replace=yes;
contree all/ majrule=yes strict=no
le50=yes
treefile = <greedyConsensusTreeFile>
replace=yes;
log stop;
quit; end;
```

2 Protocol for DACTAL-boosting

Here we describe the protocol for DACTAL-based boosting for MP-EST. Necessary scripts and softwares for this protocol are avaialable at:
`http://www.cs.utexas.edu/users/phylo/software/dcm-protocol/`

The input to DACTAL-boosting is the set of rooted gene trees $T = \{t_1, t_2, \ldots, t_k\}$ on species set S. The user must provide values for the following parameters:

- I, the number of iterations (default is $I = 5$)
- p, the padding size (default is $p = 4$)
- ms, the maximum subset size (default is $ms = 15$)

Step 1: Compute starting tree. The first step requires that the starting tree be computed. The user can select any starting tree they prefer, including one that is based on a previous estimate of the species tree for the dataset. In the paper we used two different starting trees – MRP (matrix representation with parsimony) and MP-EST.

Computing MRP starting tree:

We created MRP matrices using a custom Java program, and solved MRP heuristically using the default approach available in PAUP* (v. 4. 0b10) **?**. PAUP* generates an initial tree through random sequence addition and then performs Tree Bisection and Reconnection (TBR) moves until it reaches a local optimum. This process is repeated 1000 times, and at the end the most parsimonious tree is returned. When multiple trees are found with the same maximum parsimony score, the "extended majority consensus" of those trees is returned.

Below are the PAUP* commands used.

```
begin paup;
set criterion=parsimony maxtrees=1000 increase=no;
hsearch start=stepwise addseq=random nreps=100 swap=tbr;
filter best=yes;
savetrees file = <treeFile> replace=yes format=altnex;
contree all/ strict=yes treefile = <strictConsensusTreeFile> replace=yes;
tcontree all/ majrule=yes strict=no treefile = <majorityConsensusTreeFile>
replace=yes;
contree all/ majrule=yes strict=no le50=yes treefile = <greedyConsensusTreeFile>
replace=yes;
log stop;
quit; end;
```

Computing MP-EST starting tree:

We used version 1.3 of MP-EST. We ran MP-EST 10 times with different random seed numbers, and selected the species tree with the best likelihood score using a custom shell script.

After you have computed the starting tree, make sure it is in Newick format, and name it "starting.tre".

Step 2: Compute I different candidate species trees. Step 2 is the most complicated step, and has several sub-steps. We describe each in turn.

Step 2a: We decompose the set S of species into smaller subsets. To do this, we will use the DACTAL software, applied to the starting tree "starting.tre", and using the following command:

```
python prd_decomp.py starting.tre max_subset_size padding_size >
dactal_subsets
```

Here, `padding_size` $= n$ means n closest taxa from four subtrees around the centroid edge will be selected (so $4n$ taxa in total). Therefore, if you want the padding size to be

p, run the `prd_decomp.py` script by setting `padding_size` $= p/4$. We have provided an example output file ("dactal_subsets") of dactal decomposition in the "scripts" directory.

The output of this command (`dactal_subsets`) x subsets of taxa (one subset in each line). You should make x files ("subset_1, subset_2, subset_3, ..., subset_x") containing these x subsets, using `extract_subsets.pl` as follows. This script also creates the species lists for each of the subsets, which will be required to run MP-EST on the restricted gene trees. The command is as follows.

```
perl extract_subsets.pl -i dactal_subsets
```

Step 2b, part 1: Next we compute T_i which is the set of gene trees T restricted to the set of leaves in `subset_i`, for all $i = 1, 2, \ldots, x$.

Let "inFile" is a file containing the set T of gene trees. To compute T_i, use the script `induced_subtree_from_taxa.py` with the following command:

```
python induced_subtree_from_taxa.py inFile subset_i
```

This script will create the following files:
`inFile.subset_1, inFile.subset_2,..., inFile.subset_x`

Step 2b, part 2: For each $i = 1, 2, \ldots, x$, we estimate a species tree `speciestree_i` on `subset_i` by running MP-EST on the set T_i (`inFile.subset_i`) of rooted gene trees.
Step 2c: Combine all the trees (`speciestree_1, speciestree_2, ..., speciestree_x`), that are returned in Step 2b, part 2, in a single file called "all_sp_trees". We use SuperFine+MRL to compute the spertree on the full set of taxa from the set of species trees on the subsets of taxa. Instructions for installing SuperFine can be found at:
`http://www.cs.utexas.edu/~phylo/software/superfine/submission`
We use the following command:

```
python runReup.py -r rml -i all_sp_trees -o new_sptree
```

Save the output of this command as `new_sptree`. Repeat Step 2 for a given number of iterations (3 to 5 iterations should be enough). `new_sptree` computed in iteration i is used as the guide tree (starting tree) in Step 2a for $(i + 1)$-th iteration.

Step 3: Selecting one tree Take the list of trees you produced in Step 2c in different iterations. Score each tree with respect to the quartet support, using the script `score_tree_quartet_support.pl` as follows. (**This script requires 64 bit machine.**)

```
perl score_tree_quartet_support.pl -g inFile -s candidate_species_tree
-o score
```

Here, `inFile` contains the set T of input gene trees in Newick format (one tree in each line), `candidate_species_tree` is a species tree you want to score. The score (total number of satisfied quartets) will be saved in a file named "score".

Determine which candidate species tree, produced in Step 2c, has the largest quartet support score, and return that tree as the output of this protocol.
